# Construction of a Novel Ferroptosis-Related Gene Signature for Predicting Survival of Patients With Lung Adenocarcinoma

**DOI:** 10.3389/fonc.2022.810526

**Published:** 2022-03-03

**Authors:** Xiaojie Song, Liqun Wu, Guangqiang Wang, Baoyi Liu, Wenyong Zhu

**Affiliations:** ^1^ Department of Respiratory Medicine, Qilu Hospital (Qingdao), Cheeloo College of Medicine, Shandong University, Qingdao, China; ^2^ Department of Thoracic Surgery, Qilu Hospital (Qingdao), Cheeloo College of Medicine, Shandong University, Qingdao, China

**Keywords:** lung adenocarcinoma, ferroptosis, ferroptosis-related genes, prognostic model, risk model

## Abstract

Lung adenocarcinoma (LUAD) is the most diagnosed subtype of lung cancer; ferroptosis is widely involved in the pathological cell death associated with various cancers, including lung cancer. However, the comprehensive relationship between ferroptosis and LUAD is little known in molecular levels until now. In the present study, 513 LUAD patients could be aggregated into three clusters by consensus clustering based on RNA sequencing data of 291 ferroptosis-related genes (FRGs) in The Cancer Genome Atlas (TCGA) database; cluster2 had significant survival advantage compared to the other two clusters. A novel prognostic model of 8 differential FRGs was constructed to effectively divide LUAD patients into high- or low-risk group according to the risk scores by the Cox and LASSO regression analyses. The overall survival of LUAD patients in the high-risk group was significantly worse in the TCGA and GEO cohorts. Moreover, patients with radiation therapy or high clinical stage had obviously higher risk scores. We validated the differential mRNA and protein expression of four FRGs in paired tumor and normal samples from our clinical cohort. Our study constructed a novel FRG signature to predict the prognosis of LUAD patients, which might provide a new prognostic tool and potential therapeutic targets for LUAD.

## Introduction

Lung cancer is the most commonly diagnosed cancer and the leading cause of cancer death in the world, with 2.1 million new cases and 1.8 million deaths predicted in 2018 (1). The histological subtypes of lung cancer consist of non-small cell lung cancer (NSCLC) and small cell lung cancer (SCLC); approximately 85% of patients belong to NSCLC (2, 3). Lung adenocarcinoma (LUAD) is the most diagnosed subtype of NSCLC, followed by lung squamous cell carcinoma (LUSC) (4). LUAD is associated with distinct genomic alterations and widespread molecular heterogeneity compared with other lung cancer subtypes (4). Although substantial progress in the treatment of lung cancer have been achieved over the past decades, the 5-year survival rate of lung cancer patients remains only 4%–17% depending on stage and regional differences (5). Therefore, it is important to identify novel prognostic biomarkers and develop an effective prognostic model for predicting the survival of LUAD patients.

Historically, cell death was initially considered to be accidental and passive (6). Unlike accidental cell death, regulated cell death can be modulated through a series of cellular mechanisms and signaling pathways (7). The best-studied form of regulated cell death is apoptosis, which is mainly triggered by the activation of proteases from the caspase family (8). In recent years, there has been a growing attention in the importance of regulated cell death mechanisms beyond apoptosis in studying tumor suppression because resistance to apoptosis is a hallmark of cancer (9, 10). Ferroptosis is an iron-dependent form of regulated cell death that involves lethal, iron-catalyzed lipid damage; the term ferroptosis was described in 2012 (11). Ferroptotic death is a form of regulated cell death, as it is dramatically modulated by pharmacological perturbation of lipid repair systems involving glutathione and GPX4 (12). Since the initial description of this process, an increasing number of compounds and metabolic pathways have been identified related to ferroptosis (10, 13).

Ferroptosis has been implicated in the pathological cell death associated with various disease conditions including cancer and degenerative diseases (14, 15). Importantly, ferroptosis has potential physiological functions in tumor suppression. The p53 protein could inhibit cystine uptake and sensitize cells to ferroptosis by repressing the expression of SLC7A11, a key component of the cystine/glutamate antiporter highly expressed in human tumors (16). Similarly, tumor suppressor BRCA1-associated protein 1 (BAP1) inhibited tumor development partly through repressing SLC7A11 expression and elevating lipid peroxidation and ferroptosis (17). In LUAD, Alvarez et al. reported that suppression of NFS1 could cooperate with inhibition of cysteine transport to trigger ferroptosis *in vitro* and slow tumor growth (18). Zhang et al. found that endogenous glutamate was critical for ferroptosis sensitivity *via* ADCY10-dependent YAP suppression in LUAD, and ferroptosis-based treatment might be a good strategy for LUAD patients with later-stage and/or therapy-resistant tumors (19).

Although the expression profiles of ferroptosis-related genes (FRGs) have been utilized to develop some survival models for prognostic prediction of LUAD patients, the published studies just adopted few FRGs to analyze the prognosis signature (20–22). Therefore, we collected more FRGs to stratify LUAD patients based on mRNA expression levels in the present study. LUAD patients in three clusters had significantly distinct survival time by FRG expression signatures. Differential FRGs among the three clusters were significantly enriched in response to multiple stress- and ferroptosis-related pathways. Then, LUAD patients could be divided into high- or low-risk group according to the risk scores of the prognostic model by 8 FRGs in the training and validation cohorts. The patients with radiation therapy experience or high clinical stage had obviously high-risk scores. Our findings might be helpful to understand the potential clinical value of ferroptosis-related genes in LUAD and provide a new tool for risk and prognosis assessment in LUAD.

## Materials and Methods

### Data Collection

The RNA sequencing data, phenotype data, and corresponding clinical information of 526 LUAD patients were downloaded from TCGA database based on the Xena platform (https://xenabrowser.net/, version 07-20-2019), including 526 LUAD tumor samples and 59 normal samples. Thirteen patients did not have survival information, so the remaining 513 tumors were used for subsequent analysis. Then, all genes with zero values were removed, and the expression levels of redundant genes were averaged. In addition, genes with median absolute deviation (MAD) > 0.5 were remained, including 9,107 genes. Gene expression and clinical data of another 226 LUAD tumor samples were obtained from the Gene Expression Omnibus (GEO) GSE31210 and GSE30219 datasets. Normalized read count values were used for further analysis.

Then, 291 FRGs were retrieved from the union of the FerrDb database (http://www.zhounan.org/ferrdb, including 167 driver genes and 104 suppressor genes) and one published literature with 113 FRGs (23).

### Clinical Sample Collection

Twelve pairs of frozen tumor and matched adjacent samples of LUAD were obtained for experimental validation of differential FRGs from the Department of Thoracic Surgery in Qilu Hospital (Qingdao). All patients gave informed consent for collection of tissue collection and research testing under the supervision of the Ethics Committee of Qilu Hospital of Shandong University (Qingdao).

### Ferroptosis-Based Consensus Clustering Analysis

The number of unsupervised clusters and their stability in the TCGA LUAD dataset was estimated based on the mRNA expression profiles of 291 FRGs with the consensus clustering method *via* the ConsesusClusterPlus package in R v3.13 (http://www.bioconductor.org/).

### Differential Expression and Function Enrichment Analysis

One-way analysis of variance (ANOVA) was performed to determine significantly differential ferroptosis-related mRNAs among these three clusters. The “clusterProfiler” R package v4.0.0 was utilized to conduct Gene Ontology (GO) and Kyoto Encyclopedia of Genes and Genomes (KEGG) analyses based on the differential FRGs. The analysis threshold was determined by the adjusted p-value, and p < 0.05 indicated that the functional was significantly enriched. The edgeR and limma R packages were used to analyze expression levels of 8 FRGs in paired LUAD tumor and adjacent samples from TCGA and GEO databases, respectively. The infiltrating proportions of diverse immune cells and related genes were calculated with the single-sample gene set enrichment analysis (ssGSEA) method in the “gsva” R package (24).

### Construction and Validation of a Prognostic Ferroptosis-Related Gene Signature

A univariate Cox analysis of OS of LUAD patients was performed to screen FRGs with prognostic potential by p < 0.01. The prognostic risk signatures of 14 differential FRGs were established by using the LASSO regression analysis in the TCGA training cohort (25). Signatures were determined by selecting the optimal penalty parameter (λ) following the minimum 10-fold cross-validation. The risk scores of LUAD patients were calculated according to the normalized expression level of each FRGs and its corresponding regression coefficients. The equation was established as follows: risk score = sum of coefficients × prognostic FRGs’ expression level. According to this equation, the risk score of each patient was separately calculated in the TCGA training and GEO validation cohorts. Subsequently, the patients could be stratified into high- and low-risk groups, and the median value of the risk score was set as the cutoff point. The predictive ability of the nomogram and other predictors (age, gender, risk score, radiation therapy, EGFR mutation) for the 3- and 5-year OS was set up. Calibration curves based on the Hosmer–Lemeshow test were applied to illustrate the uniformity between the practical outcome and the model prediction outcome.

### RNA Extraction, Reverse Transcription, and qPCR

Total RNA was extracted from 12 paired tumor and adjacent samples using TRIzol Reagent (Invitrogen, Carlsbad, CA, USA). The cDNA was synthesized by Reverse Transcriptase M-MLV (Takara, Mountain View, CA, USA), and qPCR was performed with SYBR Green Dye (Applied Biosystems, Foster City, CA, USA) in triplicate. GAPDH was used as the internal control. Relative quantitation was calculated using the 2^-ΔΔCt^ method. The primers used in this study were as follows: EGLN1 5′-GCAGCATGGACGACCTGATA-3′ (F) and 5′-AGCAACCATGGCTTTCGTCC-3′ (R); MIF 5′-CATCGTAAACACCAACGTGCC-3′ (F) and 5′-CGATCTTGCCGATGCTGTG-3′ (R); PANX1 5′-GGCTGCATAAGTTTTTCCCCT-3′ (F) and 5′-GCAGCCTTAATTGCACGGTT-3′ (R); RRM2 5′-TGGTCGACAAGGAGAACACG-3′ (F) and 5′-TTAGTTTTCGGCTCCGTGGG-3′ (R); GAPDH 5′-CAGGGCTGCTTTTAACTCT GGTAA-3′ (F) and 5′-GGGTGGAATCATATTGGAACATGT-3′ (R).

### Immunohistochemical Staining

FFPE tissue blocks from 12 patients who had undergone resection were used for immunohistochemical staining (IHC) staining. Paired tumor and adjacent samples were used for staining of EGLN1 (RE6068, HUABIO, Hangzhou, China), MIF (ab65869, Abcam, Cambridge, MA, USA), PANX1 (12595-1-AP, Proteintech, Wuhan, China), and RRM2 (ab57653, Abcam). The primary antibody of EGLN1 was diluted at 1:100, others were diluted at 1:200 and incubated at room temperature, and then the secondary antibodies were added for incubation. All the staining processes were carried out on the IHC System (Roche, Basel, Switzerland) following the manufacturer’s instruction.

### Statistical Analysis

Student’s t-test and one-way ANOVA were used to conduct a difference comparison of two or three groups, respectively. The cutoff point of each group was identified by the survminer R package. Kaplan–Meier curves for OS analysis were presented between stratified subgroups with the log-rank test. Univariate and multivariate Cox regression analyses were performed to determine independent prognostic factors, which were visualized by the forestplot package in R. Operating characteristic curve (ROC) curve analyses for 1-, 3-, 5-, and all-year survival were delineated OS with R package “pROC.” All statistical analyses were carried out with the R software (version 3.5.1), and p ≤ 0.05 was considered statistically significant.

## Results

### Significant Correlation of Consensus Clustering for Ferroptosis-Related Genes With the Survival of LUAD Patients

**Figure 1:**
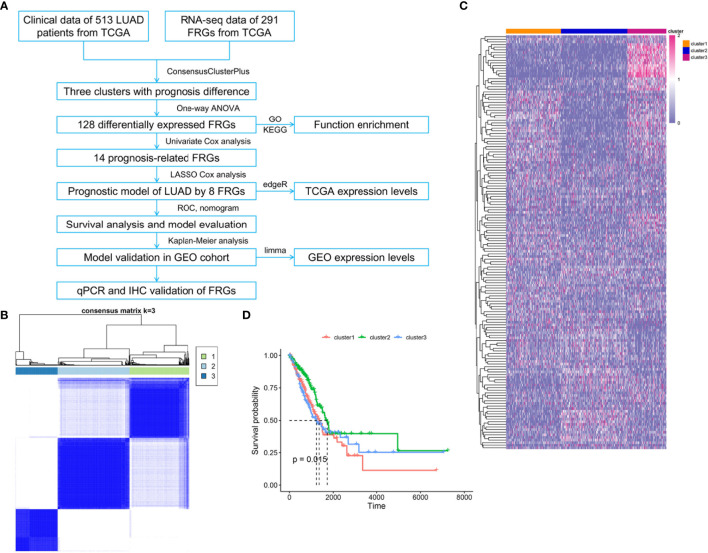
Three clusters of LUAD patients with distinct survival based on consensus clustering of FRGs. **(A)** Flowchart of data collection, analysis, and experimental validation. **(B)** Consensus clustering matrix for k = 3, LUAD patients were divided into three clusters. **(C)** Heatmap of the three clusters with different clinicopathological features based on expression levels of 291 FRGs in TCGA. **(D)** Kaplan–Meier curves of overall survival for patients with LUAD in three clusters, *p* = 0.015.

To systematically summarize this study, a workflow is shown in **Figure 1A**. Public gene expression data, phenotype, and full clinical annotation of 526 LUAD patients were obtained from the TCGA database. After filtration, 513 LUAD patients were finally enrolled with the profiles of 9,107 genes. To assess the biological functions of ferroptosis in the progression of LUAD, we investigated the expression patterns of 291 FRGs which were mainly selected from the FerrDb database. The R package of ConsensusClusterPlus was used to classify 513 LUAD patients with different expression patterns of the 291 FRGs, and k = 3 was identified with optimal clustering stability from k = 2 to 9 based on the similarity (**Figure 1B**). Eventually, three distinct clusters of LUAD patients with different clinicopathological features were identified using unsupervised clustering, including 177 cases in cluster1, 212 cases in cluster2, and 124 cases in cluster3 (**Figure 1C**). Prognostic analysis for these three main clusters revealed the prominent survival advantage in cluster2 (**Figure 1D**).

### Function Enrichment Analysis of Differential FRGs Between Three Ferroptosis Gene Clusters for LUAD

**Figure 2:**
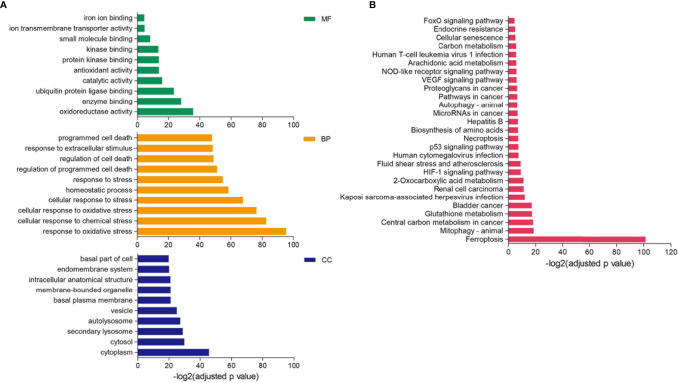
GO and KEGG enrichment analysis of differential FRGs. **(A, B)** Functional annotation of 128 FRGs using GO terms **(A)** and KEGG pathway **(B)**. “BP” stands for “biological process,” “CC” stands for “cellular component,” and “MF” stands for “molecular function,” *p*-value < 0.05.

Differential expression analysis revealed that 128 FRGs were significantly different among the three clusters of LUAD. GO enrichment showed that the biological process of these genes participated in response to multiple stress, homeostatic process, and regulation of programmed cell death. The molecular function mainly regulated oxidoreductase activity, ion transmembrane transporter activity, and iron ion binding, and cellular components were enriched in the cytoplasm, cytosol, and secondary lysosome (**Figure 2A**). The KEGG pathway analysis displayed that the differential FRGs were involved in ferroptosis, HIF-1 signaling pathway, p53 signaling pathway, and VEGF signaling pathway (**Figure 2B**).

### Construction of a Prognostic Model Based on the Ferroptosis Gene Expression Signature

**Figure 3:**
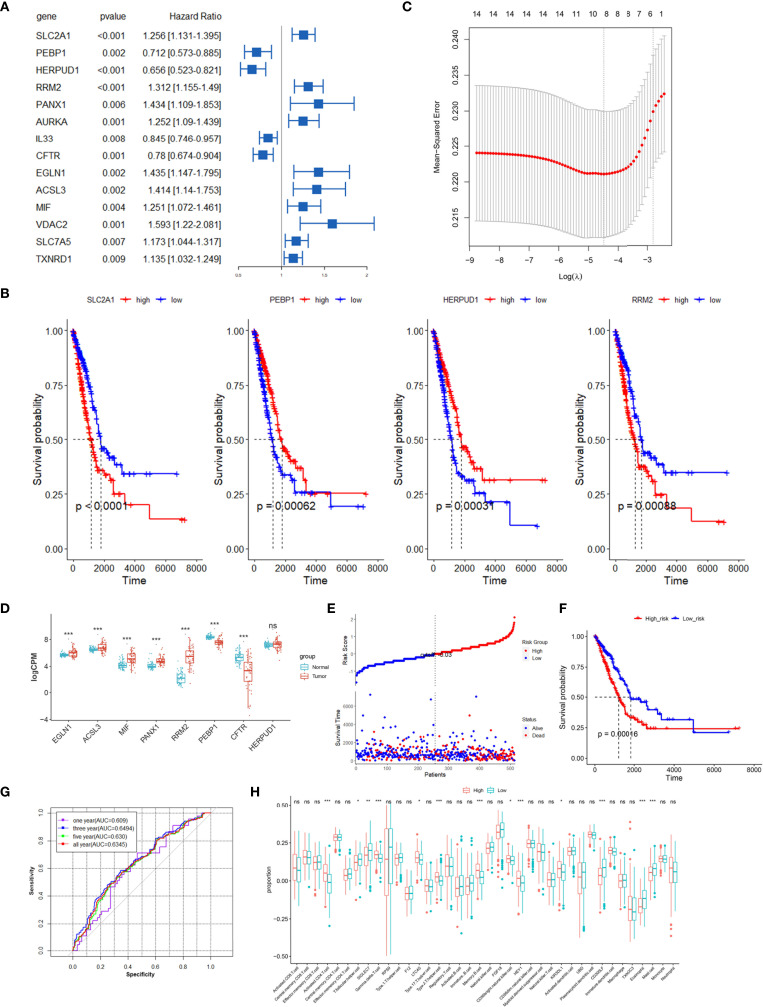
Construction prognostic signatures of FRGs in TCGA LUAD. **(A)** Univariate Cox regression analysis revealed that the 14 FRGs significantly correlated with overall survival of LUAD patients. **(B)** Survival analysis of 4 differential FRGs based on the median expression levels, another 10 FRGs are shown in **Figure S1**. **(C)** The tuning parameters (log λ) of OS-related FRGs were selected to cross-verify the error curve in LASSO regression. **(D)** Relative expression levels of 8 FRGs in paired tumor and normal samples in TCGA. **(E)** Distribution of FRG model-based risk score for the TCGA training cohort, and patterns of the survival time and survival status between the high- and low-risk groups. **(F)** Kaplan–Meier curves for the OS of LUAD patients in the high-risk group and low-risk group. **(G)** ROC curves showed the predictive efficiency of the risk signatures for 1-, 3-, 5- and all-year survival. **(H)** The infiltration proportions of diverse immune cell populations between the high-risk and low-risk groups in the TCGA LUAD cohort. *p < 0.05, **p < 0.01, ***p < 0.001, ns, not significant.

We screened ferroptosis-associated prognostic factors from the 128 differential FRGs in the TCGA training set using univariate Cox regression analysis. Fourteen FRGs in the TCGA LUAD datasets were significantly correlated with OS of LUAD patients (**Figure 3A**). Among the 14 FRGs, 12 genes could effectively predict the OS based on their median expression, respectively (p < 0.05, **Figure 3B** and **Figure S1**). SLC7A5 and TXNRD1 did not show significant prognostic associations (**Figure S1**). All 14 FRGs exhibited differential expression among the three clusters of LUAD patients (**Figure S2**).

LASSO Cox regression analysis was performed to establish a prognostic model of LUAD using the expression profiles of the 14 differential FRGs mentioned above. This method could effectively discern the most available forecast markers and produce a prognostic indicator to predict survival results. An 8-gene signature was identified based on the optimal value of λ (**Figure 3C**). Thus, we calculated the risk scores and constructed a prognostic model for the FRGs. A differential expression analysis of 57 paired samples revealed that 7 of 8 FRGs had significant differences between tumor and normal tissues (**Figure 3D**). The high expressions of EGLN1, ACSL3, MIF, PANX1, and RRM2 were correlated with poor survival of LUAD patients, and the low expressions of PEBP1, CFTR, and HERPUD1 were associated with worse survival (**Figure 3B** and **Figure S1**). The LUAD patients were stratified into a high-risk group (n = 256) or a low-risk group (n = 257) according to the median cutoff value (**Figure 3E**). The Kaplan–Meier analysis revealed that the expression of a high-risk FRG signature corresponded with poorer survival (p < 0.01, **Figure 3F**). The AUC predictive value of the eight-FRG signature for 1-, 3-, 5-, and all-year survival rate was 0.609, 0.6494, 0.630, and 0.6345, respectively (**Figure 3G**).

To further explore the correlation between immune status and the risk score in LUAD, we quantified the infiltration proportions of diverse immune cell populations with ssGSEA. The proportions of activated CD4 T cell, T follicular helper cell, type2 T helper cell, memory B cell, and mast cell were significantly different between the high-risk and low-risk groups in the TCGA LUAD cohort (**Figure 3H**). Then, we further analyzed the correlations between the infiltration proportions of immune cell and patients’ survival in LUAD and found that a high infiltration of T follicular helper cell (p = 0.02) and eosinophil (p = 0.014) had significantly better prognosis compared to the low infiltration groups (**Figure S3**). The latest research supported our conclusion about the correlations of T follicular helper cell and patients’ survival, in which neoantigen-driven CD4 T follicular helper cell and B cell collaboration promoted antitumor immunity by enhancing CD8 T cell effector functions (26).

### Independent Prognostic Value and Clinical Correlations of the 8-Gene Signature

**Figure 4:**
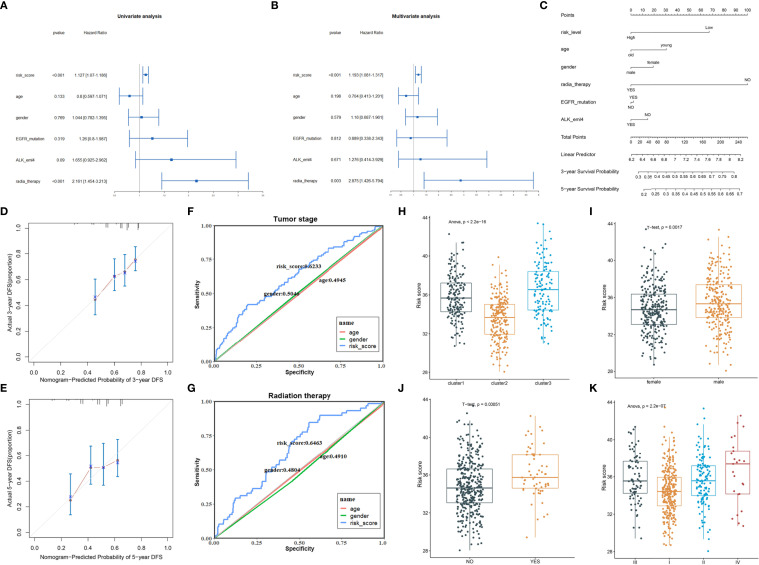
The clinical correlation analysis of the FRG prognostic model. **(A, B)** Univariate **(A)** and multivariate **(B)** Cox analysis of the clinical characteristics and risk score with the OS. **(C)** The nomogram predicted the probability of the 3- and 5-year OS. **(D, E)** The calibration plot of the nomogram predicts the probability of the 3- **(D)** and 5-year **(E)** OS. **(F, G)** ROC curves showed the predictive efficiency of the risk signatures for tumor stage **(F)** and radiation therapy **(G)**. **(H–K)** The risk scores in different clusters **(H)**, genders **(I)**, radiation therapy status **(J)**, and clinical stage **(K)**.

The univariate and multivariate Cox regression analyses were performed to evaluate whether the risk model of 8 FRGs had independent prognostic characteristics for LUAD; the results revealed that the FRG signature (HR: 1.127 and 1.193) and radiation therapy (HR: 2.161 and 2.875) were independent prognosis factors for OS of LUAD patients (p < 0.01, **Figures 4A, B**). The hybrid nomogram incorporating the FRG prognostic signature and clinicopathological characteristics was stable and accurate and thus could be applied in clinical management of LUAD patients (**Figure 4C**). The calibration curve results displayed that the observed versus predicted rates of the 3- and 5-year disease-free survival (DFS) revealed ideal consistency (**Figures 4D, E**). Moreover, the risk scores showed the better potential to predict tumor stage and radiation therapy than gender and age in LUAD (**Figures 4F, G**). Interestingly, cluster2 had a lower risk score than the other two clusters defined by ferroptosis-related genes; this verified that patients in cluster2 had survival advantage (**Figure 4H**). In addition, the male patients and patients who have undergone radiation therapy revealed higher risk scores (**Figures 4I, J**). Moreover, patients with high clinical stage had more risk scores (**Figure 4K**); these results suggested that our risk model possessed certain clinical significance based on ferroptosis-related genes.

### Validation of the 8-Gene Signature in Two GEO Datasets

**Figure 5:**
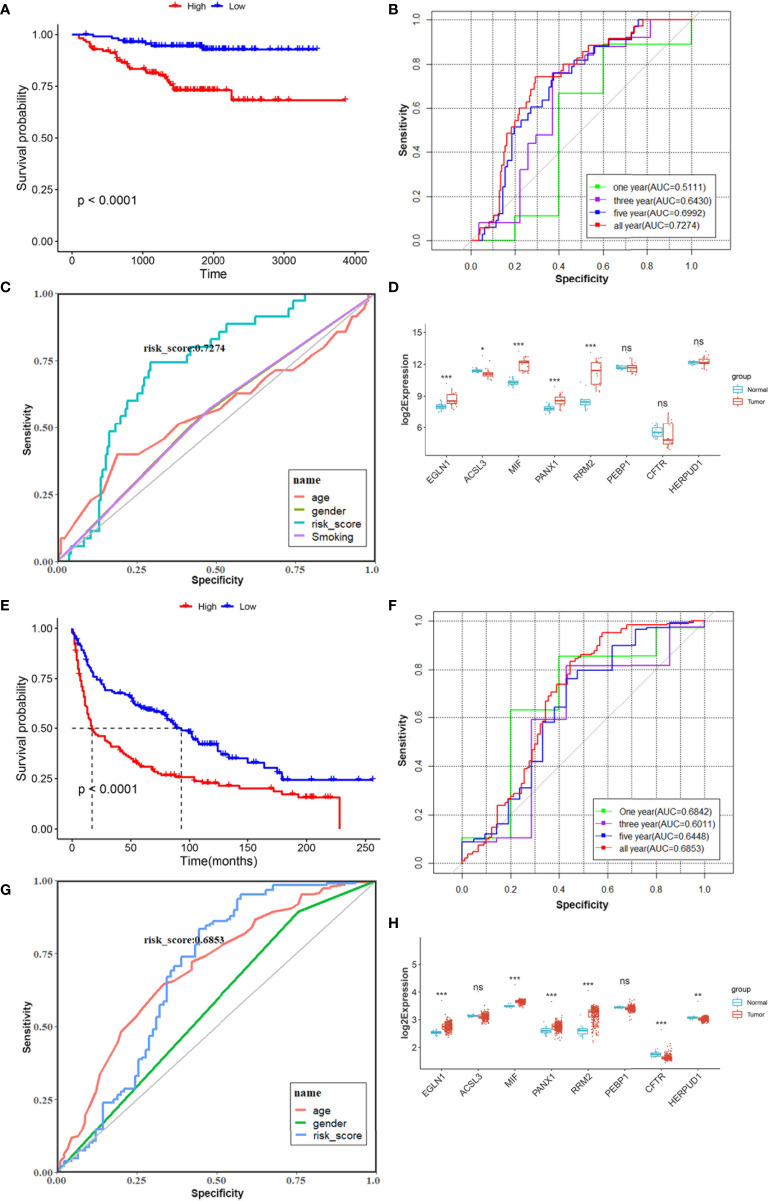
Validation prognostic signatures of FRGs in GSE31210 **(A–D)** and GSE30219 **(E–H)** cohort. **(A, E)** Kaplan–Meier curves for the OS of LUAD patients in the high-risk group and low-risk group from GEO database. **(B, F)** ROC curves showed the predictive efficiency of the risk model for 1-, 3-, 5-, and all-year survival in two GEO datasets. **(C, G)** ROC curves revealed the predictive efficiency of 8-gene signature compared with other clinical features. **(D, H)** Relative expression levels of 8 FRGs in paired tumor and normal samples. *p < 0.05, **p < 0.01, ***p < 0.001, ns, not significant.

To validate the robustness of the risk model constructed from the TCGA LUAD cohort, the 226 LUAD patients from the GSE31210 data could be also categorized into high- or low-risk groups by the same formula as that from the TCGA cohort. The high-risk group had a reduced survival time compared with those in the low-risk group (**Figure 5A**). The AUC of the 8-gene signature was 0.5111 at 1 year, 0.6430 at 3 years, 0.6992 at 5 years, and 0.7274 at all years (**Figure 5B**). Moreover, the 8-gene signature exhibited superior performance than age, gender, and smoking in predicting the prognosis of LUAD (**Figure 5C**). EGLN1, MIF, PANX1, and RRM2 showed a consistently high expression in 15 tumor samples compared to paired normal tissues (**Figure 5D**). Another dataset GSE30219 contained 278 lung cancer patients with different histological subtypes; all patients were divided into high- and low-risk groups based on the 8-FRG signature. The high-risk group showed worse prognosis (**Figure 5E**), and the AUC of the 8-gene signature was 0.6842 at 1 year, 0.6011 at 3 years, 0.6448 at 5 years, and 0.6853 at all years, respectively (**Figure 5F**). Similarly, the risk model could better predict the survival of lung cancer patients than age and gender (**Figure 5G**). EGLN1, MIF, PANX1, and RRM2 had higher expression in tumor than in normal samples (**Figure 5H**). These results suggested that the 8-gene signature of LUAD could also be used to evaluate the risk of lung cancer containing other subtypes.

### Clinical Experimental Validation of the Expression Levels of Four Differential FRGs

**Figure 6:**
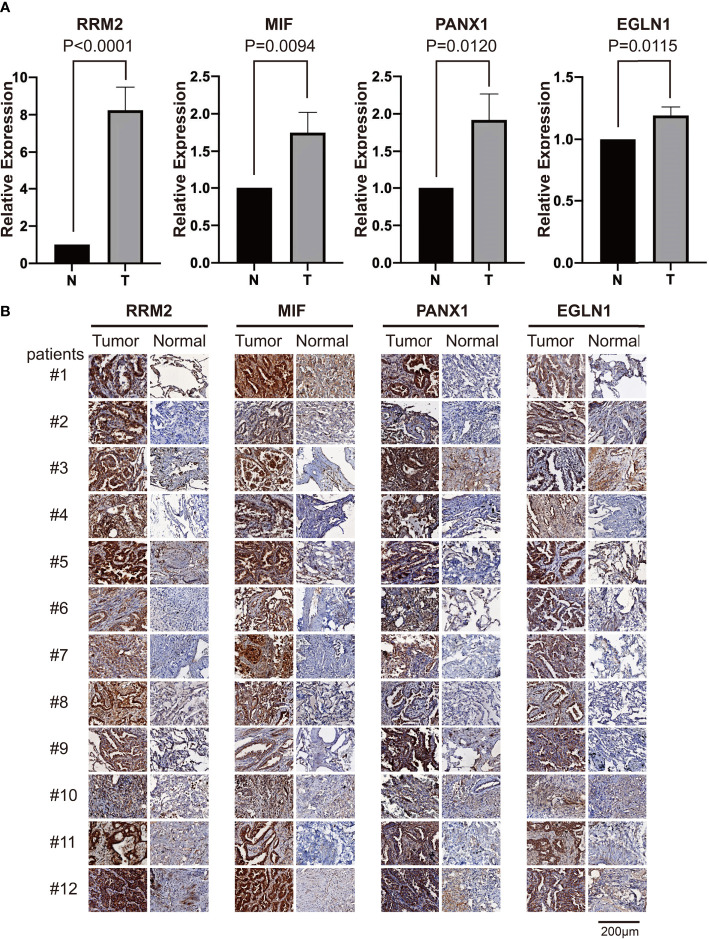
The RT-qPCR and IHC validation of relative expression levels of four differential FRGs in clinical samples. **(A)** The qPCR validation results of four differential FRGs (RRM2, MIF, PANX1, and EGLN1) in 12 paired tumor and normal samples of LUAD patients, N, normal; T, tumor. **(B)** The IHC results of these four differential FRGs in 12 paired samples of LUAD patients.

We performed the validation of mRNA and protein expression levels of 4 FRGs (RRM2, MIF, PANX1, and EGLN1) which showed a differential expression in both TCGA and two GEO datasets in our clinical specimens. The qPCR results revealed that the mRNA levels of RRM2, MIF, PANX1, and EGLN1 were significantly higher in tumor samples than in paired normal samples (**Figure 6A**). The IHC of 12 patients also showed that the protein expression levels of these 4 FRGs were higher in tumors than in corresponding normal tissues (**Figure 6B**).

## Discussion

Ferroptosis has been linked to cancer since the very beginning of this study field: the initial discovery of some chemical inducers of ferroptosis was the result of hunting for novel antitumor chemicals (27, 28). Subsequent mechanistic studies have revealed that numerous cancer-relevant genes and signaling pathways regulated ferroptosis in several types of carcinoma, including lung cancer. The FSP1–CoQ10–NAD(P)H pathway existed as an independent parallel system, which cooperated with GPX4 and glutathione to suppress ferroptosis in a number of cancer cells (29, 30). Cystine starvation of NSCLC cell lines induced accumulation of γ-glutamyl peptides, which were produced due to a non-canonical activity of glutamate-cysteine ligase catalytic subunit, and eventually limited the accumulation of glutamate, thereby protecting against ferroptosis (31). Except for some coding mRNAs, long non-coding RNA LINC00336 could also serve as an endogenous sponge of microRNA 6852 to regulate the expression of cystathionine-β-synthase, a surrogate marker of ferroptosis, and promote cell growth by inhibiting ferroptosis in lung cancer (32).

In this study, we comprehensively analyzed the clustering effects of ferroptosis genes in LUAD patients based on the expression levels of 291 FRGs. Three clusters of LUAD patients revealed a prominent prognostic difference, and a risk model of 8 FRGs was developed on the basis of differential FRGs among the three clusters. Although similar risk models of FRGs have been established in LUAD (33, 34), our analytic strategy provided a new method for discovering new prognostic-related genes in cancer by differential analysis in three clusters. The results revealed that 8 FRGs of risk models were correlated with overall survival of LUAD patients; among them, PEBP1 and ACSL3 were confirmed as prognostic factors in LUAD (21, 33, 34). However, our prognostic model identified that 6 new FRGs were significantly correlated with the survival of LUAD patients, including EGLN1, MIF, CFTR, PANX1, RRM2, and HERPUD1.

The egl-9 family hypoxia-inducible factor 1 (EGLN1) catalyzes the posttranslational formation of 4-hydroxyproline in hypoxia-inducible factor (HIF) alpha proteins (35). Recent study also showed that elevated EGLN1 expression might be a valuable biomarker of poor prognosis in patients with LUAD, but not in LUSC (36). Moreover, Reggiani et al. identified and validated that the EGLN1 gene might be a novel therapeutic target, preferentially associated with KRAS-mutated LUAD by integrating functional genomic analysis, *in vitro* data of cancer cell lines, gene druggability data, and patients’ transcriptomic and mutational data (37). Mechanistically, EGLN1 pro-oncogenic activity was partially dependent on HIF1A. The macrophage migration inhibitory factor (MIF) is a pleiotropic cytokine or growth factor that contributes to inflammatory, autoimmune, and malignant disease pathologies (38). We found that MIF was significantly upregulated in LUAD tumor tissue compared to normal samples, and a high expression of MIF was correlated with poor prognosis of LUAD patients. Kamimura also discovered that both MIF mRNA and protein were higher in LUAD specimens than in the normal alveolar epithelium (39). Winner et al. reported that a novel inhibitor that served as a suicide substrate for MIF could effectively inhibit motility and growth of lung cancer cells (38). MiR-608 could suppress LUAD invasion and migration by directly targeting MIF (40). These results suggested that MIF could be a potential prognostic biomarker and therapeutic target for LUAD. DNA methylation might be crucial for the downregulation of CFTR gene expression in lung cancer, and promoter hypermethylation of CFTR could also be an important prognostic factor in NSCLC (41). Multiple studies have shown that a high expression of RRM2 could act as an independent predictive factor of poor prognosis in LUAD patients (42, 43). Moreover, knockdown of RRM2 suppressed LUAD cell proliferation and migration *in vitro* and prolonged survival time in metastatic models (44). However, PANX1 and HERPUD1 lack related reports in LUAD except our findings in this study.

Inevitably, there are several limitations in our study. First, based on the retrospective data from the TCGA database, we constructed a prognostic model by differential FRGs to predict the survival of LUAD patients. A validation of the risk model was performed using retrospective data from the GSE31210 and GSE30219 cohorts. Thus, we need more data to verify the clinical application value of our FRG-based survival model. Second, although we analyzed the relative expression levels of 8 FRGs from this prognostic model in paired tumor and normal samples based on TCGA data; further experiments are required to validate the expression levels and risk score in clinical samples.

In conclusion, we collected FRGs and mRNA expression profiles to construct a novel risk model of 8 FRGs for predicting the overall survival of LUAD patients. This model was shown to be associated with LUAD patients’ survival and clinical stage. Our study might provide insights for further research on ferroptosis as a prognostic biomarker and potential functional target in LUAD.

## Data Availability Statement

The original contributions presented in the study are included in the article/**Supplementary Material**. Further inquiries can be directed to the corresponding author.

## Ethics Statement

The studies involving human participants were reviewed and approved by the Ethics Committee of Qilu Hospital of Shandong University (Qingdao). The patients/participants provided their written informed consent to participate in this study.

## Author Contributions

XJS designed the paper and performed bioinformatics analysis. LQW completed the data collection from the database. GQW completed the data verification and partial information analysis. BYL completed the immunohistochemical experiment. WYZ critically reviewed and edited the manuscript. All authors contributed to the article and approved the submitted version.

## Funding

This work was supported by the Shandong Province Key Research and Development Program (2016GSF201102) and the Scientific Research Start-up Foundation of Shandong University Qilu Hospital (Qingdao) (QDKY2019YC05 and QDKY2019ZD01).

## Conflict of Interest

The authors declare that the research was conducted in the absence of any commercial or financial relationships that could be construed as a potential conflict of interest.

## Publisher’s Note

All claims expressed in this article are solely those of the authors and do not necessarily represent those of their affiliated organizations, or those of the publisher, the editors and the reviewers. Any product that may be evaluated in this article, or claim that may be made by its manufacturer, is not guaranteed or endorsed by the publisher.

## Supplementary Material

Supplementary Figure 1 Survival analysis of another 10 differential FRGs based on the median expression levels, related to **Figure 3B**.

Supplementary Figure 2 The relative expression levels of 14 differential FRGs among the three clusters, related to **Figure 3**.

Supplementary Figure 3 The correlations between the infiltration proportions of immune cell and patients’ survival in LUAD, related to **Figure 3H**.
