# Supplementary figures and images for: Construction of a Novel Ferroptosis-Related Gene Signature for Predicting Survival of Patients With Lung Adenocarcinoma
Source: Front Oncol. 2022 Mar 3;12:810526. doi: 10.3389/fonc.2022.810526 (PMC8928751; doi:10.3389/fonc.2022.810526)

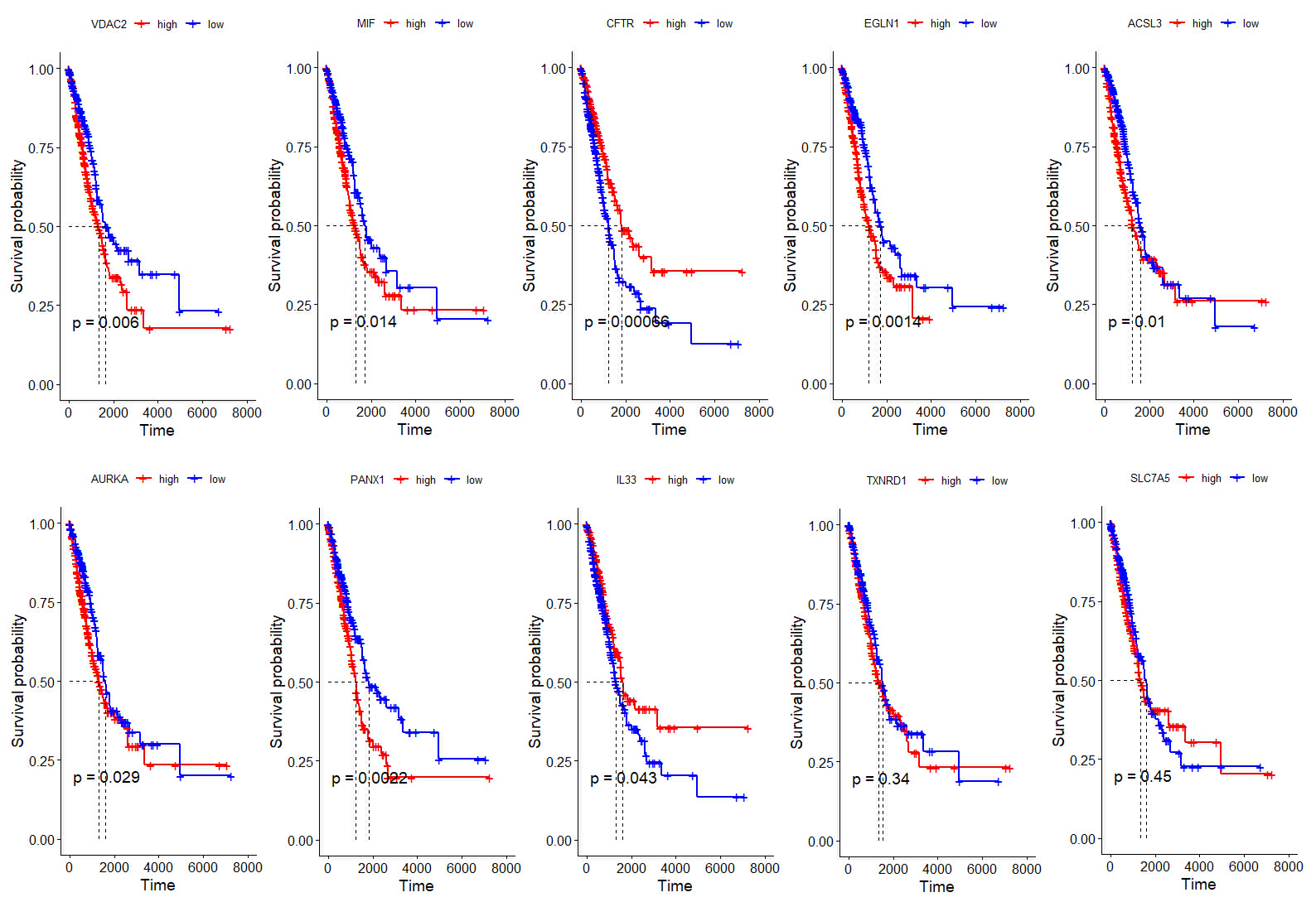

Supplement: Supplementary Figure 1 — Survival analysis of another 10 differential FRGs based on the median expression levels, related to Figure 3B . [file Image_1.tif]

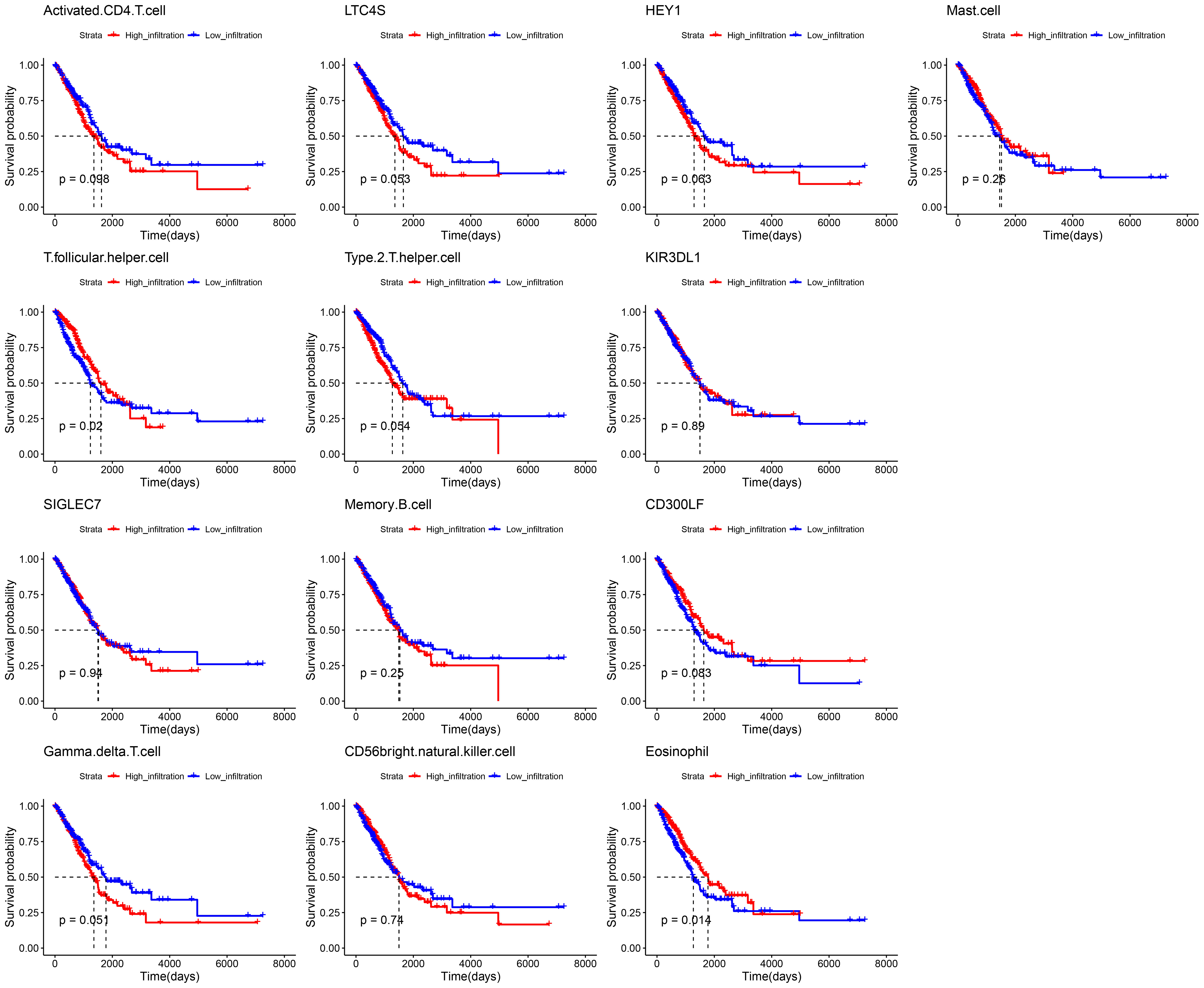

Supplement: Supplementary Figure 3 — The correlations between the infiltration proportions of immune cell and patients’ survival in LUAD, related to Figure 3H . [file Image_3.tif]
